# Prevalence and antimicrobial resistance of *Salmonella* and pathogenic *E. coli* in broiler farms, Wakiso district, Uganda

**DOI:** 10.1371/journal.pone.0309599

**Published:** 2025-07-01

**Authors:** Thomas Ssemakadde, Nalumaga Pauline Petra, Jude Collins Busingye, Joel Bazira, Kabanda Taseera

**Affiliations:** Department of Microbiology, Faculty of Medicine, Mbarara University of Science and Technology, Mbarara, Uganda; Fayetteville State University, UNITED STATES OF AMERICA

## Abstract

**Background:**

The emergence and re-emergence of zoonotic bacterial infections and the upsurge reflected in current trends of antimicrobial-resistant bacteria is a major global concern. *Salmonella* spp and *Escherichia coli (E. coli)* are the two most important food-borne pathogens of public health interest incriminated in poultry products worldwide. AMR in poultry farming poses a significant public health risk in Uganda, as the misuse and overuse of antibiotics in livestock can lead to the emergence of resistant pathogens that may transfer to humans through direct contact, consumption of contaminated poultry products, or environmental exposure, further complicating the management of infection hence necessitating constant monitoring of microbial food safety measures.

**Methods:**

This study was a cross-sectional study that used a total of two hundred sixteen poultry samples from cloacae swabs and fecal swabs collected from broiler poultry farms. These were cultured on Chromagar ^TM^ *Salmonella* and Sorbitol MacConkey agar. Biochemical tests, minimum inhibitory concentration, and polymerase chain reaction were utilized. Data was analyzed by descriptive statistics, and Chi-square (χ²) Test statistical significance of quantitative data.

**Results:**

A total of 40 (18.5%) *Salmonella* and 120 (55.6%) pathogenic *E. coli* were isolated while extended beta-lactamase (ESBL) production was detected in 18 *Salmonella* and 57 pathogenic *E. coli* isolates. Prevalence of *bla*TEM gene was expressed in 7/18 (39%) *Salmonella* isolates and 42/57 (73.8%) Pathogenic *E. coli* isolates The significant associated factors that predisposed these farms to *Salmonella* spp was source of poultry feeds (p-value = 0.066) while factors associated with pathogenic *E. coli* included contact of poultry with other birds and livestock (p-value = 0.020), movement from one pen to the other by farm-handlers (p-value = 0.017), use of untreated water (p-value = 0.018) and food contamination of commercial poultry feeds (p-value = 0.0021).

**Conclusion:**

The findings of this study highlight the significant presence of *Salmonella* and pathogenic *E. coli* in poultry farms, underscoring the potential risks to public health. The high prevalence of antimicrobial resistance observed among these isolates calls for urgent interventions to curb the misuse of antibiotics in poultry farming.

## Introduction

Globally poultry farming is one of the fast-growing low-cost investment ventures currently on the rise. This is partly explained by the significant growth in the population size that provides a ready market for poultry products [1]. As a result, birds are raised in rigorous, intense circumstances using antimicrobial drugs to hasten growth and prevent disease due to the method of raising the birds [2]. Antibiotic-resistant bacteria in poultry could have resistant genes that could potentially be passed on to humans posing threats such as economic losses in animal production and treatment failure, leading to mortalities and morbidities [3].

According to the World Health Organization, the global burden associated with consumption of ASFs at 168 Disability adjusted life years (DALYs) per 100,000 population (equivalent to 35% of the global burden of Food Borne Diseases) [4].Furthermore, World Bank estimated the cost of unsafe food in Africa to be 16.7 billion US dollars, comprising productivity and treatment losses [4].

Antimicrobial resistance (AMR) in Uganda’s poultry farming sector is a growing public health concern, driven by the overuse and misuse of antibiotics [5]. Many studies have highlighted high rates of resistance in common pathogens like *E. coli* and *Salmonella* spp, with significant resistance to antibiotics such as tetracycline, amoxicillin, and ciprofloxacin [5–7]. Factors such as inadequate knowledge among farmers, poor biosecurity practices, and widespread antibiotic use contribute to the problem [7]

In Wakiso District, Uganda, the poultry market has grown rapidly due to changing consumer preferences and a high demand for fast food products, particularly broilers, which offer quick returns on investment. Uganda Bureau of Standards (UBOS 2018) estimates the current poultry population to be above 35.4 million poultry birds. According to the census, the Wakiso District area produced 7.4% of the chicken consumed by the nation and was ranked as the top producer of poultry goods (MAAIF and UBOS, 2010).

According to disease surveillance and outbreak data published in the Ministry Of Health weekly Epidemiological bulletin, there is an upsurge in the number of enteric diseases such as typhoid seen in Kampala and Wakiso districts [8]. The existence of these food-borne pathogens in poultry leads to poses a significant public health risk in Uganda, as the misuse and overuse of antibiotics in livestock can lead to the emergence of resistant pathogens that may transfer to humans through direct contact, consumption of contaminated poultry products, or environmental exposure, further complicating the management of these infections [9]. Owing to the upward increase in the human population and increased enteric illnesses, there is a need to investigate whether these poultry products play a part in perpetuating these infections. Addressing AMR in poultry farming stricter antibiotic regulations, and enhanced infection control measures to protect both animal and human health. This study therefore determined the antimicrobial susceptibility patterns, related variables, and prevalence of *Salmonella* and pathogenic *E. coli* in poultry farms in Wakiso District to provide detailed information on the extent of spread and resistant genes.

## Materials and methods

### Study area

This study was conducted within selected counties of Wakiso district a metropolitan district in the central region of Uganda that partly encircles Kampala district. The district coordinates are 00 24N, 32 29E. With a projected population of 2,915,200 million, Wakiso district is still Uganda’s most populous Higher Local Government (HLG) [10]. The population is projected to grow at 4.1% annually, which increases the demand for food, particularly poultry products. The district was regarded as the top producer of poultry, producing 7.4% of all the chickens in the country [11].

### Study design

This was a cross-sectional study focused on broiler farms from August to September 2021 based on the Food and Agriculture organization (FAO) categorization sector level 3 because they are more representative of the commercial poultry farming sector, which involves in larger-scale operations with higher antibiotic usage compared to small-scale farms [12].

### Sample size determination

The sample size was determined using Kishi-Leslie (1965) formula using 83% prevalence [13].


$$n=\frac{Z^{2}P(1-P)}{d^{2}}$$

n = study sample size required

Z= critical value associated with 95% confidence interval = 1.96

P = Estimated prevalence of 83%of *E. coli* and *Salmonella* d = margin of error = 0.05


$$n=\frac{1.96^{2}(0.83\times 0.17)}{0.05^{2}}$$

n = 216 samples

Therefore, 216 farms within Wakiso District were considered. The farm managers of the farms were interviewed to understand the associated factors that predispose these pathogens with consent.

### Sample collection

Simple random sampling with the selection process stratified by size, and production system, to select the sub-counties was employed while purposive sampling used the information provided by the district veterinary office to identify the villages. For the selected and eligible farms three swabs from fresh fecal droppings and two swabs from the cloaca were collected from randomly selected old (5–7weeks) birds [14]. Five swabs from one farm on different chickens were considered to represent one sample (Pooling of samples). Strict biosecurity measures were followed during sampling to ensure data integrity and minimize contamination risk. Personnel wore appropriate personal protective equipment including gloves and masks, which were changed between farm visits. Samples were collected using sterile swabs to prevent cross-contamination. The swabs were placed into a plain labeled tube containing Amie’s transport media without charcoal and transported to the laboratory within 24 hours to maintain their integrity.

### Isolation and identification of microorganisms

The pooled samples were aseptically mixed into 9 ml of autoclaved Buffered Peptone Water (BPW) (Hi Media M1494, Mubi, India) in a sterile 50 ml falcon tube with a lid to generate a pre-enriched sample and incubated for 16–24 hours at 37°C aerobically.

The enriched sample was picked and inoculated onto a Chromagar^TM^ of *Salmonella* designed for the presumptive identification, qualitative direct detection, and differentiation of *Salmonella* species [15]. Plates were incubated at 37°C for 24 hours. Presumptive colonies for *Salmonella* (mauve-pink, raised, and smooth colonies) were selected for identification.

For isolation of pathogenic *E. coli*, the pre-enriched sample was inoculated on Sorbitol MacConkey agar plates and incubated at 37°C for 24 hours. The colonies of pathogenic *E. coli* were smooth, raised, had entire margins, and were colorless on SMAC.

The presumptive organisms were sub-cultured on nutrient agar and identified through gram staining, Triple Iron Sugar (TSI) test, the IMViC test, Methyl Red, Voges- Proskauer, Sulphur indole motility, and Simmons citrate utilization. All reagents used were from (Oxoid, England) [16] and *Escherichia coli* (ATCC 25922) was used as the control strain.

### Bacterial antibiotic susceptibility testing

Bacterial suspension was adjusted to 0.5 McFarland standard and inoculated on Muller Hinton agar medium (Oxoid CM0337 Basingstoke, England) using surface spreading method. The antibiotic discs listed in Table 1 (all from Oxoid, England) were used. The plates were incubated for 24 hours at 37°C. The results were read and interpreted according to Clinical Laboratory Standards Institute, 2020 [17]. *E. coli* ATCC 25922 (American Type Culture Collection, Rockville, MD, USA) was used as a reference control strain.

**Table 1:** Commonly used antibiotics at NaLIRRI (National Livestock Resources Research Institute) Microbiology laboratory, obtained from Oxoid Ltd suppliers, and the discs were selected based on CLSI (28^th^ Edition, 2020).

Antibiotic Class	Antibiotics used for both *E. coli* and *Salmonella* isolates
Aminoglycosides	Gentamicin (GM) (10 *μ*g/ml)
Carbapenem	Meropenem (MEM) (10 *μ*g/ml)
Cephalosporins class III	Ceftriaxone (CRO) (30 *μ*g/ml)
Amphenicol	Chloramphenicol (CHL) (30 *μ*g/ml)
Cephalosporins class IV	Cefepime (CPM) (30 *μ*g/ml), Cefotaxime (CTX)(30 µg/ml), Ceftazidime (CTZ) (30 µg/ml)
Quinolones	Ciprofloxacin (CIP) (5 *μ*g/ml)
Macrolides	Erythromycin (EM) (30 *μ*g/ml)
Penicillin	Ampicillin (AMP) (10 *μ*g/ml)
Tetracyclines	Tetracycline (OXT) (30 *μ*g/ml)
Sulphonamides/ Trimethoprim	Co-trimoxazole (CXT) (25 *μ*g/ml)

### Phenotypic screening of enterobacteriaceae for extended-spectrum β-lactamases (ESBLs) production

Extended-spectrum β-lactamases (ESBLs) are enzymes with the ability to hydrolyze third-generation cephalosporins but are inhibited by clavulanic acid [17].

A sterile cotton swab was used to surface spread the bacterial suspension onto a Mueller Hinton agar plate. The antibiotic discs used were Ceftazidime (CAZ) (30 μg) alone and Ceftazidime in combination with Clavulanic acid (CAL) (30/10 μg) [18]. The discs were spaced approximately 30 mm apart. The plates were incubated at 37° C overnight. Both the single disc and the combined disc’s zones of inhibition were measured. The results were interpreted according to CLSI guidelines [18].

### Genomic DNA extraction

Bacterial genomic DNA extraction was conducted on all 18 *Salmonella* and 57 pathogenic *E. coli* following manufacturer instructions of the Bio line ISOLATE II genomic DNA kit (Cat No. Bio-52065 Lot No. IS502-B054750). The presence of genes encoding for ESBL (*bla*TEM gene) was detected using conventional PCR amplification using primers listed in (Table 2).

**Table 2:** Primers used in the PCR reaction.

Resistance Gene	Primer Sequence (5′ → 3′ Direction)	Amplicon Size (bp)	Annealing Temperature (°C)	Reference
*bla*_TEM_	(F) TGG GTG CAC GAG TGG GTT AC	526	58	Tenover et al., 1994
	(R) TTA TCC GCC TCC ATC CAG TC			

The PCR master Mix reagents were prepared by mixing 12.5 µL master mix consisting of One Taq quick load two times master mix/w standard buffer, dNTPs and Taq polymerase (M0486S), 1.5 µL forward (100 µM), 1.5 µL primary reverse (one hundred µM), and5 µL DNA template and RNAse-free dH2O up to 25 µL.

The PCR process was carried out in a thermocycler (Perkin Elmer, Wellesley, MA, USA) with a pre-denaturation cycle of 95°C for 15 min, followed by a DNA amplification stage with 30 cycles (94°C for 1 min, 58°C for 1 min, and 72°C for 1 min) and final extension cycle of 72°C for 5 min.

DNA Amplicons were electrophoresed using 1.5% agarose gel, in Tris-Borate EDTA buffer (TBE) 1 × concentration, Safe View Classic^TM^ DNA stain, 6x loading dye (Thermo Scientific), and DNA ladder/marker 100 bp (Sigma-Aldrich, Inc., Saint Louis, MI, USA) DNA Bands were visualized on a Dark reader Transilluminator.

### Data management and analysis

All records of the analysis were recorded in the lab register as a hard copy backup, and securely stored. Samples were assigned codes and Excel spreadsheets were used to enter the raw data, which were then exported to Stata (Version 12, Special Edition, College Station, Texas USA) for analysis. Double entry of data was done to rule out any errors. Frequency tables and graphs were used to present descriptive statistics. Chi-square (χ²) Test determined the variables of statistical significance of Quantitative data.

### Ethical consideration

Approval was obtained from Mbarara University of Science and Technology; the Institutional Ethical Review Committee (MUST-2021-141), and at the ministry level, the permanent secretary Ministry of Agriculture, Animal Industries, and Fisheries, the district’s chief administrative officer, and the district veterinarian.

Before engaging the farms, we obtained verbal consent from the farm owners to access their farms and collect samples. Data and bacterial isolates obtained for this investigation were handled with confidentiality.

## Results

### Demographic and socio-economic characteristics of the respondents

Of the 216 samples collected, a total of 40 (18.5%) *Salmonella* species and 120 (55.6%) pathogenic *E. coli* species were isolated. The study involved farm managers from ten (10) different sub-counties in Wakiso district, with a total of 216 participants interviewed. Among them, 87 (40.28%) were male, and 129 (59.72%) were female as seen in Table 3.

**Table 3:** Farm characteristics and demographics.

Characteristics	Category	Frequency (N = 216)	Percentage
(Variable)			(%)
Gender	Male	87	40.28
	Female	129	59.72
Age (years)	15–30	53	24.54
	31–45	113	52.31
	>=46	50	23.15
Education level	Primary	58	26.85
	Secondary	80	37.04
	Vocational Training	33	15.28
	University	45	20.83
Sub counties	Kitabi	25	11.57
	Namugongo	28	12.96
	Kiira	32	14.81
	Makindye	28	12.96
	Nabweru	19	8.80
	Kakiri	21	9.72
	Busukuma	11	5.10
	Wakiso TC	16	7.41
	Masulita	12	5.56
	Nsangi	24	11.11
Source of income	Poultry alone	146	67.59
	Livestock farming (cattle, goats, sheep, or pigs) with poultry	22	10.19
	Crop farming with poultry	17	7.87
	Self-employed off-farm (others)	31	14.35
Hygiene/rate of cleaning of the farm	Once per month	13	6.02
	Once per week	117	54.17
	Daily	86	39.81
Type of production system used	Deep litter	103	47.70
	Battery cage system	82	37.96
	Mixed farming with other birds like turkeys and ducks	19	8.77
	Semi-intensive (Free range system)	12	5.57

### Factors associated with *Salmonella* and pathogenic *E. coli* in broiler poultry farms in Wakiso District

*Salmonella* and pathogenic *E. coli* were most frequently found in broiler poultry farms where there was contact between livestock and other bird species like turkeys and geese (p-value = 0.020), use of untreated water (p-value = 0.018) contamination of commercial poultry feeds (p-value = 0.002) (Table 4).

**Table 4:** Factors associated with *Salmonella* species and *E. coli* in poultry farms in Wakiso District.

Variable	Category	*Salmonella*		*E. coli*	
		Frequency n (%)	p-value	Frequency n (%)	p-value
Contact of livestock and other bird species	Livestock (cattle, goats, sheep, or pigs)	83 (69.2)	0.384	65 (81.25)	0.020
	Other birds (geese, ducks, turkeys)	25 (20.8)		15 (18.75)	
Frequency of cleaning and disinfection of the bird’s housing.	Daily	96 (80)	0.498	49 (61)	0.113
	Once a week	21 (17)		26 (33)	
	Once a month	03 (3)		05 (6)	
Contamination of commercial poultry	Mixed with water	95 (79.2)	0.242	56 (70)	0.002
	Mixed with other materials	25 (20.8)		24 (30)	
Movement from one pen to the other by farm-handlers	From one pen	58 (48.3)	0.244	55 (68.75)	0.017
	From more than one pen	62 (51.7)		25 (31.25)	
Use of untreated water	Use of well water	38 (31.7)	0.392	32 (40)	0.018
	Use of tap water	82 (68.3)		48 (60)	
Source of poultry feeds	Home made	46 (38.3)	0.066	25 (31)	0.237

### Antibiotic susceptibility patterns of *Salmonella* and pathogenic *E. coli* towards commonly used antimicrobials in poultry

*Salmonella* species isolated from broiler poultry samples were highly resistant to ampicillin 32 (80%). On the contrary, low resistance was observed to and meropenem n = 1 (2.5%) as shown in Table 5.

**Table 5:** Antibiotic susceptibility patterns of *Salmonella* isolates from poultry.

Antibiotics	Resistant n (%)	Intermediate Resistance n (%)	Susceptible n (%)
Ampicillin (AMP) (10 μg/ml)	32 (80)	2 (5)	6 (15)
Erythromycin (EM) (30 μg/ml)	28 (70)	7 (17.5)	5 (12.5)
Tetracycline (OXT) (30 μg/ml)	27 (67.5)	6 (15)	7 (17.5)
Ciprofloxacin (CIP) (5 μg/ml)	25 (62.5)	10 (25)	5 (12.0%)
Sulfamethoxazole-trimethoprim (SXT) (25 μg/ml)	20 (50)	5 (12.5)	15 (37.5)
Ceftriaxone (CRO) (30 μg/ml)	18 (45)	4 (10)	18 (45)
Ceftazidime (CTZ) (30 µg/ml)	15 (37.5)	5 (12.5)	20 (50)
Cefotaxime (CTX)(30 µg/ml)	10 (25)	2 (5)	28 (70)
Chloramphenicol (CHL) (30 μg/ml)	3 (7.5)	3 (7.5)	34 (85)
Gentamicin (GM) (10 μg/ml)	3 (7.5)	7 (17.5)	30 (75.0)
Cefepime (CPM) (30 μg/ml)	2 (5.0)	3 (7.5)	35 (87.5)
Meropenem (MEM) (10 μg/ml)	1 (2.5)	2 (5.0)	37 (92.5)

*E. coli* exhibited the highest resistance to erythromycin at 88 (73%), followed by ampicillin at 86 (72%). However, very low resistance to cefipime 3 (2.5%) is seen in Table 6.

**Table 6:** Antibiotic susceptibility patterns of pathogenic *E. coli* from poultry.

Antibiotics	Resistant n (%)	Intermediate Resistance n (%)	Susceptible n (%)
Erythromycin (EM) (30 μg/ml)	88 (73)	15 (12.5)	17 (14.5)
Ampicillin (AMP) (10 μg/ml)	86 (72)	12 (10)	18 (15)
Chloramphenicol (CHL) (30 μg/ml)	85 (71.0)	25 (21.0)	10 (8.0)
Tetracycline (OXT) (30 μg/ml)	82 (68.0)	18 (15)	20 (17.0)
Sulfamethoxazole-trimethoprim (SXT) (25 μg/ml)	78 (65)	32(27.0)	10 (8.0)
Ceftriaxone (CRO) (30 μg/ml)	57 (47.5)	5 (4.2%)	58 (48.3)
Cefotaxime (CTX)(30 µg/ml)	51 (42.5)	10 (8.3)	59 (49.2)
Ceftazidime (CTZ)(30 µg/ml)	48 (40)	8 (6.7)	64 (53.3)
Gentamicin (GM) (10 μg/ml)	10 (8.0)	7 (6.0)	103 (86.0)
Ciprofloxacin (CIP) (5 μg/ml)	9 (7.5)	66 (55)	45(37.5)
Meropenem (MEM) (10 μg/ml)	4 (3)	6 (2.5)	110 (92.0)
Cefepime (CPM) (30 μg/ml)	3 (2.5)	8 (6.5)	109 (91)

### Detection of ESBL (blaTEM), a gene encoding resistance to commonly used antibiotics used in poultry in *Salmonella* and pathogenic *E. coli*

The *bla*TEM gene was expressed in 7 of the 18 (39%) *Salmonella* and 42 of the 57 (73.8%) *E. coli* isolates respectively.

## Discussion

The prevalence of *Salmonella* and pathogenic *E. coli* found in our research study was comparable with a study carried out at Makerere University by Kakooza and colleagues [5] that reported the prevalence of *Salmonella* and pathogenic *E. coli* as 21.1% and 56.3% in Uganda. However, the prevalence in our present study is low when compared to a study done by Mjalija and colleagues who found an overall prevalence of 83%, of which 90.8% and 73% were from chicken in Lira and Kampala districts from the antibiotic susceptibility profiles of *fecal E. coli* isolates [19].This discrepancy can be due to the different study sites, sample methods, poultry sector, and sampling times used during the research.

A lower prevalence of *Salmonella* spp was isolated compared to other studies in East Africa such as one done by Dorica et al [20] in Ruiru Sub-County, Kenya which was at 28% with almost similar prevalence of pathogenic *E. coli* of 58%. In another study on antimicrobial resistance in *Salmonella* and *E. coli* isolates from chicken droppings in Nairobi, Lydia et al [21] reported a lower prevalence (12%) of *Salmonella* spp and a similar prevalence of *E. coli* (57%) in the analyzed samples. The differences in environmental contamination levels, poultry management practices, breed, sample size, sampling, testing methodologies, and challenges in *Salmonella* detection methods may account for this similar trend of reduced *Salmonella* isolation [22].

East African countries have adopted various policies to address antimicrobial resistance (AMR), but differences in implementation and surveillance capacity may influence AMR trends. Uganda’s National Action Plan (NAP) on AMR (2018–2023) focuses on public awareness, infection prevention, antimicrobial stewardship, surveillance, and research [23]. Kenya’s NAP (2017–2022) aligns with the WHO’s Global Action Plan and integrates a One Health approach, supported by national and county-level antimicrobial stewardship committees such as the Kenya Accreditation society [24]. Tanzania has developed a robust AMR surveillance framework covering both human and animal health sectors [25]. At a regional level, the East African Community (EAC) has introduced a policy framework to harmonize antibiotic supply and production among the countries [26]. While these initiatives reflect strong commitments to tackling AMR, variations in enforcement and resource availability could contribute to differing resistance patterns across the region.

Unfortunately, vaccines exist only in private practitioners’ clinics and cannot be accessed freely by the Biosecurity level three farmers that were of interest in this study in Uganda.

Interestingly, co-resistance mechanisms play a significant role in the persistence and spread of antimicrobial resistance [27]. Exposure to heavy metals such as copper, and zinc, commonly added to poultry feed for growth promotion and disease prevention, has been linked to the selection of antibiotic-resistant bacteria [27,28]. Genes conferring resistance to these metals are often located on the same mobile genetic elements as antibiotic resistance genes, facilitating the co-selection of multidrug-resistant strains [29].

The study further investigated factors associated with proper poultry practices such as the implementation of strict bio-security interventions such as visitors having restricted access by farmers, implementation of a solid well-established sewer system. These, however, did not significantly increase the risk of *Salmonella* and *E. coli*. This is comparable to a study conducted in Nigeria that focused on the risk factors to *Salmonella* spp. the presence of rodents, farm workers moving between pens, running and parking trucks close to poultry farms (p < 0.05), and drinking untreated water (p < 0.05) were all independently associated with a higher risk of *Salmonella* infection [30].

Broilers are known for consuming large amounts of feed, and this habit encourages constant feces loss, raising the possibility of their environment becoming contaminated with various bacterial strains [5].Furthermore, many broiler farms had high stock densities, which could make environmental management efforts to reduce bacteria in the houses more difficult. As a result, workers (especially those handling large flocks) must be strictly supervised because it is claimed that they may neglect their responsibilities for maintaining hygiene. Therefore, poor management of poultry could lead to increased transmission of *Salmonella* and *E. coli* in poultry.

The rapid spread of antibiotic resistance has been primarily attributed to the inappropriate and excessive use of antibiotics in various sectors, such as agriculture, specifically in livestock production systems [31,32]. Antibiotics are frequently employed in livestock farming to prevent disease, promote growth, and treat infections [7,33]. However, AMR patterns vary across livestock types, with Multidrug resistant *E. coli* more prevalent in poultry [32], while *Staphylococcus* spp. and *E. coli* in pigs exhibit high resistance to penicillin and sulfamethoxazole [34]. Additionally, poultry farmers tend to use antibiotics more for prophylaxis and promote growth [7,35], while cattle keepers primarily use them for treatment [33].

This inappropriate use of antibiotics creates selective pressure, promoting the survival and proliferation of ESBL-producing bacteria [36]. ESBL genes are located on plasmids that can be easily transferred between different bacterial species through horizontal gene transfer. In poultry, where bacteria are in close contact due to the high density of birds, this gene transfer is more frequent. Furthermore, poultry gut microbiota is highly diverse, providing ample opportunity for resistant and non-resistant bacteria to interact, increasing the likelihood of gene transfer [37,38].

The prevalence of the *bla*TEM gene in *E. coli* and *Salmonella* from poultry in Uganda aligns with findings from previous studies in the country and the broader East African region. Research conducted in Kenya and Tanzania identified prevalence of 21% and 10% of *bla* TEM gene respectively [39,40]. These findings indicate that antimicrobial resistance in poultry is a growing concern in across East Africa, mirroring trends observed in neighboring countries such as Kenya and Tanzania.

Majority of studies on poultry across the globe have also noted the presence of ESBL encoding genes such as; *bla*TEM, and AmpC [35,41–46]. In Spain [47] and Egypt [48], poultry isolates of *E. coli* carrying *blaTEM* contribute significantly to antimicrobial resistance in foodborne pathogens, while in parts of Asia, excessive antibiotic use in poultry farming has been linked to the emergence and spread of blaTEM-harboring bacteria [49]. This is a major public health concern due to its potential zoonotic transmission to humans through direct contact, consumption of contaminated products, or environmental exposure [48,50].

Given these findings, strengthening surveillance programs, enforcing regulations on antibiotic use in poultry as well as feed additives, exploring alternative disease control measures such as vaccines and probiotics are critical steps in mitigating the spread of *bla*TEM-mediated resistance in Uganda’s poultry sector and the entire livestock sector.

However, study has some limitations. Our focus was solely on broiler poultry about to enter the food chain, which does not provide a complete picture of the disease burden in poultry. Other avian species, such as layers, geese, guinea fowls, and ducks, were not included, limiting our understanding of disease transmission dynamics. Additionally, potential biases arising from the farm selection process and self-reported data from farm managers should be acknowledged, as these factors may have influenced the completeness of the information collected. A broader investigation covering all bird species would be necessary to inform more effective policies for controlling the spread. Future studies could address biases by incorporating more diverse farm sources. Furthermore, this study focused only on the *bla*TEM gene, limiting the scope of genetic analysis. Future research should explore additional ESBL genes, such as *bla*CTX-M and *bla*SHV, through various genetic manipulations to provide a more comprehensive understanding of resistance mechanisms

## Conclusion

The findings of this study highlight the significant presence of *Salmonella* and pathogenic *E. coli* in poultry farms, underscoring the potential risks to public health. The high prevalence of antimicrobial resistance observed among these isolates calls for urgent interventions to curb the misuse of antibiotics in poultry farming.

Policies should be implemented by the government of Uganda to regulate the use of antimicrobials in poultry production, promoting responsible antibiotic stewardship. Strengthening biosecurity measures, enhancing farmer education on antimicrobial resistance, and encouraging the adoption of alternative disease prevention strategies, such as probiotics and vaccination, are essential. Regular surveillance and monitoring of AMR patterns in poultry farms should also be prioritized to inform effective control strategies as well as integrating veterinary, environmental, and public health sectors, to mitigate the spread of resistant bacterial strains and ensuring food safety.
